# Electrohydrodynamic (EHD) drying of the Chinese wolfberry fruits

**DOI:** 10.1186/s40064-016-2546-1

**Published:** 2016-06-28

**Authors:** Maosheng Yang, Changjiang Ding

**Affiliations:** Physical Experiment Center, College of Science, Inner Mongolia University of Technology, Hohhot, Inner Mongolia China

**Keywords:** Electrohydrodynamic drying, Chinese wolfberry fruits, Drying rate, Rehydration ratio, Mathematical models

## Abstract

The conventional methods of drying Chinese wolfberry fruits cause loss of active ingredients and the drying time is very long. In order to explore and investigate the new method of drying Chinese wolfberry fruits, electrohydrodynamic (EHD) drying system was used to drying for Chinese wolfberry fruits with a multiple needle-to-plate electrode on five levels alternating voltage at 0, 20, 24, 28 and 32 kV and a multiple needle-to-plate electrode on a level direct voltage at 28 kV. The drying rate, the moisture rate, shrinkage rate, rehydration ratio, and Vitamin C contents of Chinese wolfberry were measured. Ten different mathematical drying models were also determined and compared to simulate drying curves based on the root mean square error, reduced mean square of the deviation and the coefficient of correlation. Each drying treatment was carried out at (25 ± 2) °C, the drying relative humidity was (30 ± 5) % and all samples were dehydrated until they reached the final moisture content (17 ± 1)/100 g. The results showed that the drying rate of Chinese wolfberry was notably greater in the EHD system when compared to control, and improved by 1.8777, 2.0017, 2.3676 and 2.6608 times, respectively, at 20, 24, 28 and 32 kV, compared to that of the control in the 5 h. The drying rate with multiple needles-to-plate electrode under AC electric field is faster than that with a multiple needle-to-plate electrode under DC electric field and the mass transfer enhancement factor heightened with the increase of voltage. The EHD drying treatments have a significant effect on rehydration ratio, and Vitamin C contents of Chinese wolfberry, but no significant differences was observed in shrinkage rate of Chinese wolfberry. The specific energy consumption of EHD drying (kJ·kg^−1^ water) were significantly influenced by the alternating voltage, it heightened with the increase of voltage. The Parabolic model was best suited for describing the drying rate curve of Chinese wolfberry fruits. Therefore, this work presents a facile and effective clue for experimentally and theoretically determining the EHD drying properties of Chinese wolfberry.

## Background

The Chinese wolfberry is distinguished herb in traditional Chinese medicine. Its ripe fruits contain massive nourishing substances which include 18 kinds of amino acids, 8 kinds of essential amino acids and 21 kinds of trace minerals, including, for example, zinc, copper, calcium, germanium, selenium, phosphorus, and take on sanative active ingredient such as polysaccharides, flavonoids, carotenoids, superoxide dismutase (SOD), alkaloids, etc. (Zhao et al. 2015; Liu et al. 2014). The fresh wolfberry fruits deteriorate rapidly after harvesting, and the dried wolfberry is more popular because of longer shelf life and significant reduction in the volume of the product. Currently, many drying techniques have been applied to the Chinese wolfberry, among them solar drying (He and Bin 2006), hot air (Wu et al. 2015), and microwave (Ma et al. 2015). These drying methods cause loss of active ingredients and the drying times are very long. Recently, some advanced drying technologies were reported for the Chinese wolfberry fruits, such as vacuum drying (Wang et al. 2000), and spray drying (Rong et al. 2013). However, these methods have also some disadvantages, such as complex equipment, large investment, and high energy consumption, which hindered the industrial applications of these methods. Consequently, growing interest has been shown in exploring new drying methods for the Chinese wolfberry.

Electrohydrodynamic (EHD) drying is a novel non-thermal technique that has been developed (Basiry and Esehaghbeygi 2010; Esehaghbeygi and Basiry 2011; Bai et al. 2012). Isobe et al. (1999) indicated that the drying rate of agar gel under the EHD system was about 3 times faster than the control. Hashinaga et al. (1999) found that application of EHD accelerated the initial drying rate of apple slices by 4.5 times over the ambient air drying. In a separate study, some authors conducted a set of EHD experiments on okara cake (Li et al. 2006), sea cucumber (Bai et al. 2013), Japanese radish (Bajgai and Hashinaga 2001b), wheat (Cao et al. 2004a), mushroom slices (Dinani and Havet 2015), carrot slices (Alemrajabi et al. 2012), etc., and found that the drying rate under EHD system is greater than the control. Bajgai and Hashinaga (2001a) found that ascorbic acid contents in spinach were substantially higher after EHD drying than after oven drying. Ding et al. (2015) demonstrated that the application of EHD contributed toward an increase by 11.53 % in the carotene contents of dried carrots, compared to oven drying. Bai et al. (2013) found that sea cucumbers dried by EHD displayed much higher protein values, which is more than that of those dried oven. Compared to hot air drying, some studies found that EHD drying offers superior quality in terms of such physiochemical properties as color, shrinkage, and nutrient content (Dinani et al. 2015). To the best of our knowledge, no studies have reported on the drying of Chinese wolfberry using EHD.

Consequently, some researchers have investigated some semi-theoretical and empirical models to describe the EHD drying process of different products. For instance, Li et al. (2005) found that the drying kinetics of okara cake with electric field best suited Page’s model. Cao et al. (2004b) studied the effect of EHD drying on rough rice, and found that the moisture content (%) of rough rice against time in an electric field treatment was described satisfactorily by an exponential model. Bai et al. (2011) studied the thin layer EHD drying of fish, and found that the quadratic model described satisfactorily the drying rate curve of fish in an EHD dryer. Six different mathematical drying models were compared to simulate EHD drying curves of beef by Ding et al. and it was found that the Demir et al. model was the best mathematical model (Ding et al. 2014). The drying process is concerned with the texture of the materials, as well as the drying technology, and, as such, it is very complex. Therefore, it is equally difficult to propose an appropriate mathematical model to describe the drying process.

The main goal of this paper was to report on the use of EHD for drying Chinese wolfberry fruits, considering drying rate, quality and mathematical models. To accomplish this, we studied the electric parameters, the drying rate and the dried quality of Chinese wolfberry fruits using EHD, including, for example, the remaining moisture content, shrinkage, rehydration ratio, Vitamin C contents. Ten mathematical models were compared to determine which one best represented the drying characteristics of the Chinese wolfberry fruits. The Chinese wolfberry fruits have undergone a systematic and comprehensive investigation in relation to EHD processing.

## Materials and methods

### Experimental equipment

**Fig. 1:**
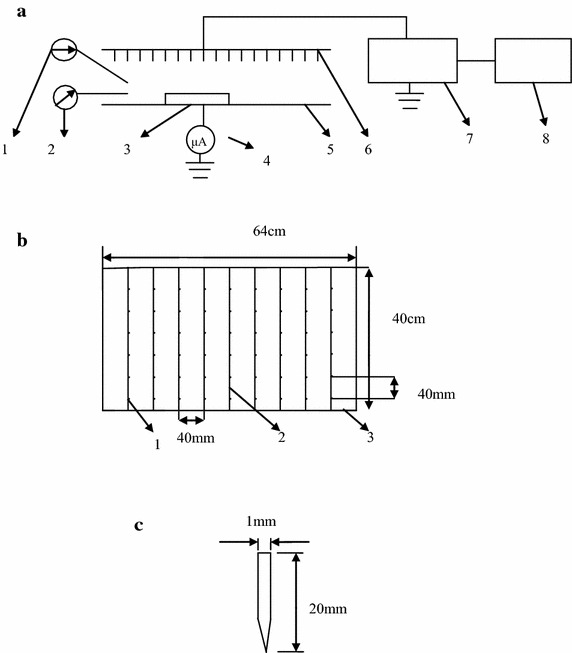
**a** Schematic diagram of EHD drying. *1* Thermometer. *2* Hygrometer. *3* Sample. *4* Amperemeter. *5* Grounded electrode plate. *6* Needle electrode. *7* High voltage power source. *8* Voltage regulator. **b** Arrangement diagram of needle electrodes. *1* Needle electrodes. *2* Stainless steel wire. *3* Stainless steel frame. **c** Schematic diagram of needle electrodes

Figure 1a shows a schematic diagram of the experimental installation for EHD drying. It is composed of a vertically mounted electrode with multiple sharp pointed needles or a flat plate projected onto a fixed horizontal grounded metallic plate upon which Chinese wolfberry fruits to be used for drying experiments were flatted. The distance between the emitting point and the grounded electrode was 100 mm to facilitate the calculation of electric field intensity. The upper electrode plate were connected to a power source able to supply alternating current (AC) high voltage or direct current (DC) high voltage with both negative and positive polarity. In order to set the desired high voltage parameters for EHD drying, the power was connected to a voltage regulator, with an adjustable voltage ranging from 0 to 50 kV for alternating current (AC) or 0–70 kV for direct current (DC) by a controller. The top flat plate was a 40 cm × 34 cm rectangular stainless steel plate and the grounded plate electrode was an 84 cm × 44 cm rectangular stainless steel plate. Figure 1b, c shows the arrangement and schematic diagrams of the needle electrodes, respectively. The needles were 20 mm long with a diameter of 1 mm. The distance between two needle electrodes was 40 mm. We enhanced water evaporation with multiple needles-to-plate electrode under AC electric field to judge relationship of between the distance of two needle electrodes and drying rate and energy consumption. Through a small factorial design experimentation, we found that if the distance of two needle electrodes was wider than optimum distance, the drying rate was lower than that of optimum distance, the energy consumption was less than that of optimum distance, as the distance became wide, the drying rate decreased, while energy consumption reduced. In addition, if the distance of two needle electrodes was narrower than optimum distance, the drying rate was also lower than that of optimum distance, but the energy consumption was more than that of optimum distance, Hence, the distance was found to be superior to the others based on the correlation between drying rate and energy consumption. The needle electrodes were arranged in multiple rows and lined up by a stainless steel wire. The distance between two stainless steel wires was 40 mm. All the samples were randomly spread in a single layer on the grounded electrode plate. The frequency of the electric field was 50 Hz.

### Determination of initial moisture content

The Chinese wolfberry fruits were purchased from a local farm (tuoketuo county, Hohhot, China). The Chinese wolfberry fruits were picked and then stored in a refrigerator at 4 °C. The initial moisture content of the Chinese wolfberry fruits was measured by a rapid moisture tester (Sh10A, Shanghai Luheng Instrument Co., Ltd., Shanghai, China). The initial moisture content of Chinese wolfberry fruits was (69 ± 1) %.

### Drying experiment

The Chinese wolfberry fruits were removed from the refrigerator and recovered to the ambient temperature naturally. 50 g of the Chinese wolfberry fruits samples were immersed in 300 mL of 5 % sodium carbonate solution at ambient conditions for 10 min. Then they were got out from sodium carbonate solution and removed excess water. The samples were put into the experimental EHD system for two different experiments. One is changed voltage each time at 0, 20, 24, 28 and 32 kV with multiple needles-to-plate electrode for AC electric field, respectively. The other is changed experimental conditions each time with multiple needles-to-plate electrode under AC electric field, multiple needles-to-plate electrode under DC electric field, the voltage is 28 kV. The mass of sample in the drying process was measured by a Sartorius Analytical Balance BS124S (Goettingen, Germany) every 1 h. The drying temperature was (25 ± 2)  °C. The drying relative humidity was (30 ± 5) %. A natural convection process was assumed in drying process. Experiments were independently performed three times in this study. All data are expressed as mean ± standard deviation (SD).

Moisture content and moisture ratio in the drying process is defined as

1 $$m_{g} = m_{0} \times (1 - M_{0} )$$

2 $$M_{i} = \frac{{m_{i} - m_{g} }}{{m_{g} }} \times 100\;\%$$

3 $${\text{MR}} = \frac{{M_{i} - M_{e} }}{{M_{0} - M_{e} }}$$

where *M*_0_ is the initial moisture content of Chinese wolfberry fruits, *M*_*e*_ is the equilibrium moisture content of Chinese wolfberry fruits, *M*_*i*_ is the moisture content of Chinese wolfberry fruits (g water/g dry matter) at time *t*_i_, *m*_0_ is the initial mass of Chinese wolfberry fruits, *m*_*i*_ is the mass of Chinese wolfberry fruits at time *t*_*i*_ and *m*_*g*_ is the dry matter of Chinese wolfberry fruits.

The drying rate (DR) was calculated using the following equation (Dinani and Havet 2015):

4 $${\text{DR}} = \frac{{M_{t} - M_{{t +\Delta t}} }}{{\Delta t}}$$

where *M*_*t*_ is moisture content of Chinese wolfberry fruits at *t*, *M*_*t*+Δ*t*_ is moisture content of Chinese wolfberry fruits at *t* + Δ*t* (g water/g dry matter), *t* is time (h).

The mass transfer enhancement factor (the evaporation enhancement factor) and Sherwood number (Sh) was calculated using the following equation (Lai et al. 2004):

5 $${\text{MTEF}} = \frac{{{\text{Sh}}_{kV} }}{{{\text{Sh}}_{0} }}$$

6 $${\text{Sh}} = \frac{{h_{m} d}}{D} = \left[ {\frac{{\dot{m}}}{{A_{c}\Delta c}}} \right]\frac{d}{D}$$

7 $$c = \frac{{0.622\rho P_{g} }}{{P - \varphi P_{g} }}$$

8 $$\Delta c = c_{0} - c_{\infty }$$

where MTEF is the mass transfer enhancement factor, Sh_kv_ is the Sherwood number with EHD, Sh_0_ is the Sherwood number without EHD, *h*_*m*_ is the mass transfer coefficient, *d* is diameter of the needle electrode (m), *D* is mass diffusivity (m^2^·s^−1^), $\dot{m}$ is mass transfer rate (g·s^−1^), *A*_*c*_ is surface area of sample that is exposed to corona wind (m^2^), *c* is the water vapor concentration, *c*_0_ is water vapor concentration at the sample surface (kg·m^−3^), *c*_*∞*_ is water vapor concentration in air (kg·m^−3^), Δ*c* is the difference in water vapor concentration between the sample surface and ambient, *ρ* is density of air (kg·m^−3^), *φ* is relative humidity, *P* is atmospheric pressure (kPa), *P*_*g*_ is saturated pressure of water vapor (kPa).

### Shrinkage rate

The volumes of both fresh and dried Chinese wolfberry fruits of each drying treatment for multiple needles-to-plate electrode under AC electric field were measured by the displacement method, in which distilled water was used as the reference liquid. Shrinkage rate (SR) was determined using the following equation (Dinani and Havet 2015):

9 $${\text{SR}} = \frac{{V_{0} - V_{f} }}{{V_{0} }} \times 100\;\%$$

where SR is the shrinkage rate of the Chinese wolfberry fruits (%), *V*_0_ is the volume of the Chinese wolfberry fruits before drying (cm^3^), *V*_*f*_ is the final volume of the Chinese wolfberry fruits at the end of drying (cm^3^). Experiments were independently performed three times in this study and an average taken.

### Rehydration ratio

To calculate the value rehydration ratio (RR) of dried Chinese wolfberry fruits of each drying treatment for multiple needles-to-plate electrode under AC electric field, 3 g of dried Chinese wolfberry fruits were immersed in 25 mL distilled water at ambient conditions for 7 h. The temperature of the distilled water was (37 ± 1) °C. The Chinese wolfberry fruits were weighed after removing excess water with the help of absorbent paper. RR was determined using the following equation (Ding et al. 2015).

10 $${\text{RR}} = \frac{{m_{a} }}{{m_{b} }}$$

where *m*_*a*_ is the weight of the dried Chinese wolfberry fruits after rehydration, *m*_*b*_ is the weight of dried Chinese wolfberry fruits before rehydration. This experiment was independently repeated three times under the same conditions and an average calculated.

### Determination of Vitamin C

Content of Vitamin C in Chinese wolfberry fruits were determined by the iodometry. 1.23 g of crushed Chinese wolfberry fruits were put into 1 mL HAc solution and 2 mL standard starch solution. Then they were put into 100 cm^3^ boiling distilled water and evenly mixed. They were titrated with iodine solution until the colour of solution turned into light blue. The percentage of Vitamin C in Chinese wolfberry fruits was calculated using the following equation:

11 $$W_{{{\text{C}}_{6} {\text{H}}_{8} {\text{O}}_{6} }} \left( \% \right) = \frac{{C_{{I_{2} }} V_{{I_{2} }} M_{{{\text{C}}_{6} {\text{H}}_{8} {\text{O}}_{6} }} }}{m} \times 100\;\%$$

where $W_{{{\text{C}}_{6} {\text{H}}_{8} {\text{O}}_{6} }}$ is the percentage of Vitamin C in Chinese wolfberry fruits, $c_{{I_{2} }}$ is the concentration of iodine solution,

$V_{{I_{2} }}$ is the volume of iodine solution, *m* is mass of the Chinese wolfberry fruits. This experiment was independently repeated three times under the same conditions and an average calculated.

### The specific energy consumption

The specific energy consumption is defined as amount of energy needed to evaporate unit mass of water in kJ·kg^−1^. To calculate the value the specific energy consumption of the Chinese wolfberry fruits of each drying treatment for multiple needles-to-plate electrode under AC electric field, the samples were dried until they reached the final moisture content (17 ± 1)/100 g. The specific energy consumption for the EHD drying (SEC_EHD_) was determined in kJ·kg^−1^ using the following equation (Dinani and Havet 2015; Martynenko and Zheng 2016):

12 $${\text{SEC}}_{\text{EDH}} = \frac{V \times I}{{m_{0} - m_{t} }} \times\Delta t$$

where *V* is voltage (kV), *I* is the current of ionic wind (μA), *m*_0_ is the weight of the samples at the beginning of the drying experiment (kg), *m*_*t*_ is the weight of the samples reached the final moisture content (17 ± 1)/100 g, Δ*t* is the time (h).

### Statistical analysis

Single-factor analysis of variance was used to calculate the drying rate and moisture ratio between the Chinese wolfberry fruits under alternating electric field and without electric field (control). The drying rate and moisture ratio between different types of electric field and electrodes were also calculated using single-factor analysis of variance. The differences in moisture ratio are considered statistically significant when (*p* < 0.05).

Shrinkage rate, rehydration ratio and content of Vitamin C were also calculated between the Chinese wolfberry fruits treated under EHD drying and the control using single-factor analysis of variance. The results reported in this study are presented as mean ± standard deviation (SD).

### Mathematical model

The number of thin-layer drying-curve models has more than 60 (Kucuk et al. 2014). Table 1 shows the ten different semi-theoretical and empirical models which had been applied to describe the drying kinetics in EHD drying (Dinani et al. 2014). The model best suited for describing the drying rate curve of Chinese wolfberry fruits was selected based on the values of the statistical parameters at 20, 24, 28 and 32 kV for AC electric field, respectively.

**Table 1:** Mathematical models applied to the drying curves

Model name	Model equation	References
Lewis (Newton)	${\text{MR}} = e^{ - kt}$	Liu et al. (2009)
Henderson and Pabis	${\text{MR}} = ae^{ - kt}$	Shen et al. (2011)
Logarithmic	${\text{MR}} = ae^{ - kt} + b$	Shahhoseini et al. (2013)
Parabolic (polynomial)	${\text{MR}} = a + bt + ct^{2}$	Bai et al. (2011)
Page	${\text{MR}} = e^{{ - kt^{n} }}$	Li et al. (2005)
Dinani et al.	${\text{MR}} = a\exp \left( { - \left( {\frac{t - b}{c}} \right)^{2} } \right)$	Dinani et al. (2014)
Wang and Singh	${\text{MR}} = 1 + at + bt^{2}$	Kaleta and Górnicki (2010)
Modified Page	${\text{MR}} = \exp \left( { - (kt)^{n} } \right)$	Akpinar and Bicer (2008)
Midilli et al.	${\text{MR}} = a\exp \left( { - kt^{n} } \right) + bt$	Midilli et al. (2002)
Weibull	${\text{MR}} = \exp \left( { - \left( {\frac{t}{b}} \right)^{a} } \right)$	Puente-Díaz et al. (2013)

### Statistical parameters

The non-linear regression analysis was implemented in order to estimate the constants and parameters of the mathematical model. The root mean square error (E_RMS_), reduced mean square of the deviation (*χ*^2^) and the coefficient of correlation (*R*^2^) were used as the primary criteria to select the equation that best accounts for the variation in the drying curves of the dried samples. Higher *R*^2^ value and lower E_RMS_ and *χ*^2^ values were chosen as the criteria for goodness of fit (Jena and Das 2007; Kiranoudis et al. 1997; Kaleta and Górnicki 2010; Mengesa and Ertekin 2006; Darabi et al. 2001). E_RMS_ gives the deviation between the predicted and experimental values. *R*^2^ also gives predictive power of the model relative to the drying behavior of the product, and its highest value is 1. These statistical values can be calculated as follows (Dinani et al. 2014; Ding et al. 2015):

13 $${\text{E}}_{\text{RMS}} = \sqrt {\frac{1}{N}\sum\limits_{i = 1}^{N} {\left( {{\text{MR}}_{{{\text{pre,}}i}} - {\text{MR}}_{{{\text{exp,}}i}} } \right)^{2} } }$$

14 $$\chi^{2} = \frac{{\sum\nolimits_{i = 1}^{N} {\left( {{\text{MR}}_{{{\text{exp,}}i}} - {\text{MR}}_{{{\text{pre,}}i}} } \right)^{2} } }}{N - n}$$

15 $$R^{2} = \frac{{\sum\nolimits_{i = 1}^{N} {\left( {{\text{MR}}_{i} - {\text{MR}}_{{{\text{pre,}}i}} } \right) \cdot \sum\nolimits_{i = 1}^{N} {\left( {{\text{MR}}_{i} - {\text{MR}}_{{{ \exp },i}} } \right)} } }}{{\sqrt {\left[ {\sum\nolimits_{i = 1}^{N} {{\text{MR}}_{i} - {\text{MR}}_{{{\text{pre,}}i}}^{2} } } \right] \cdot \left[ {\sum\nolimits_{i = 1}^{N} {\left( {{\text{MR}}_{i} - {\text{MR}}_{{{ \exp },i}} } \right)^{2} } } \right]} }}$$

where MR_exp,*i*_ is the *i*th experimental moisture, MR_pre,*i*_ is the *i*th predicted moisture, *N* is the number of observations, *n* is the number of constants in the drying model, and *i* is the number of terms.

## Results and discussion

### Drying rate

**Fig. 2:**
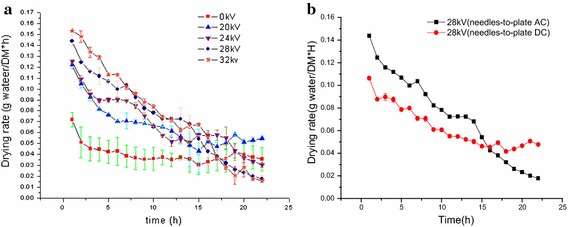
Variation of drying rate of Chinese wolfberry fruits. **a** Different voltages, **b** different electrodes

**Fig. 3:**
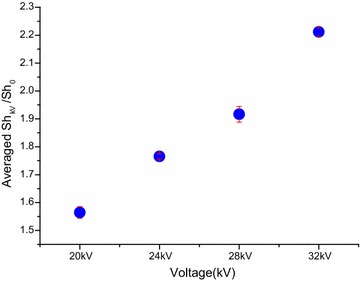
Comparison of the mass transfer enhancement factor versus different applied voltage

Figure 2a shows that the drying rate curve of Chinese wolfberry fruits treated with different voltage values at 0, 20, 24, 28, 32 kV with multiple needles-to-plate electrode under AC electric field, respectively. This figure depicts that no constant rate period was surveyed in the EHD process. The drying rate increased under the EHD process with increasing electric field ranged from 20 to 32 kV. In addition, the drying rate of Chinese wolfberry fruits exposed to EHD process at four levels of voltages was higher than that of the control in the 17 h and improved by 1.8777, 2.0017, 2.3676 and 2.6608 times, respectively, at 20, 24, 28 and 32 kV, compared to that of the control in the 5 h. These results agree with those studies which reported enhancement in drying rate with increase of applied voltage (Ding et al. 2015; Basiry and Esehaghbeygi 2010). The principal mechanism accounted for EHD drying is the production of corona wind by applying a high voltage between two electrodes with substantially different radii of curvature (Dinani et al. 2014), the impingement of this wind on wet materials has produced an impact and thus, enhances mass transfer rates of water through increased turbulence (Esehaghbeygi et al. 2014). We calculated the drying ratio for Chinese wolfberry under alternating electric field and without electric field using single-factor analysis of variance. The drying rate values showed statistically significant difference at 24 kV (*p* < 0.05) and very statistically significant difference at 28 and 32 kV (*p* < 0.01) compared to control. The mass transfer enhancement factor (the evaporation enhancement factor) versus different alternating voltage for the EHD drying system at 20, 24, 28, 32 kV with multiple needles-to-plate electrode under AC electric field are shown in Fig. 3. By ANOVA, the results indicates that there was very significant difference in terms of the mass transfer enhancement factor among 20, 24, 28, 32 kV treatments (*p* < 0.001). It is very clear that the mass transfer enhancement factor heightened with the increase of voltage. This relationship is in consistent with the consequence discovered by Lai and Wong (2003) and Lai et al. (2004) who reported that in the absence of cross-flow, the mass transfer enhancement factor strengthened with the voltage increase. Heidarinejad and Babaei (2015) reported that when the cross-flow velocity was 1.1 m s^−1^, it causes significant evaporation enhancement with the voltage increase, in other word the mass transfer enhancement factor (the evaporation enhancement factor) significantly enhanced with the increase of voltage.

Figure 2b shows that the comparison of drying rate of Chinese wolfberry treated by EHD at 28 kV with multiple needles-to-plate electrode under AC electric field and multiple needles-to-plate electrode under DC electric field. This figure depicts that the Chinese wolfberry treated at 28 kV with multiple needles-to-plate electrode under AC electric field has the higher average drying rate than that of the Chinese wolfberry treated at 28 kV with multiple needles-to-plate electrode under DC electric field. The average drying rate ratios of Chinese wolfberry treated by EHD with multiple needles-to-plate electrode under AC electric field for 5 h increases by 1.40 times, compared to that with multiple needles-to-plate electrode under DC electric field. The AC electric field performs better than DC electric field under the same voltage. In other words, the type of electric field had a most significant effect on enhancing the drying rate. This phenomenon can be illuminated from Table 2. The current versus voltage for the EHD drying system at 28 kV multiple needles-to-plate electrode under AC electric field and multiple needles-to-plate electrode under DC electric field was quite different (Table 2). The value of 28 kV multiple needles-to-plate electrode under AC electric field treatment to 28 kV multiple needles-to-plate electrode under DC electric field treatment was as high as 36.9 times. The enhanced corona wind velocity by electric field can be expressed as function of the corona current, when other conditions were fixed, the corona wind velocity enhance with the increase of corona current and the corona wind can enhanced water evaporation rate (Lai et al. 2004; Heidarinejad and Babaei 2015). As can observed from Table 2, the mass transfer enhancement factor of 28 kV multiple needles-to-plate electrode under AC electric field treatment is very more bigger than that of 28 kV multiple needles-to-plate electrode under AC electric field treatment, so Fig. 2b can account for this phenomenon which the AC electric field performs better than DC electric field under the same voltage. In other words, the type of electric field had a most significant effect on enhancing the drying rate. We calculated the drying ratio for Chinese wolfberry under each drying conditions using single-factor analysis of variance. The drying rate values showed statistically significant difference (*p* < 0.05) for each drying conditions.

**Table 2:** The mass transfer enhancement factor and current versus voltage for the EHD drying system at 28 kV of the two types of voltages

Type of voltage	28 kV (needles-to-plate DC)	28 kV (needles-to-plate AC)
Current (μA)	10	369
The mass transfer enhancement factor	1.16033	1.96314

### Moisture ratio

**Fig. 4:**
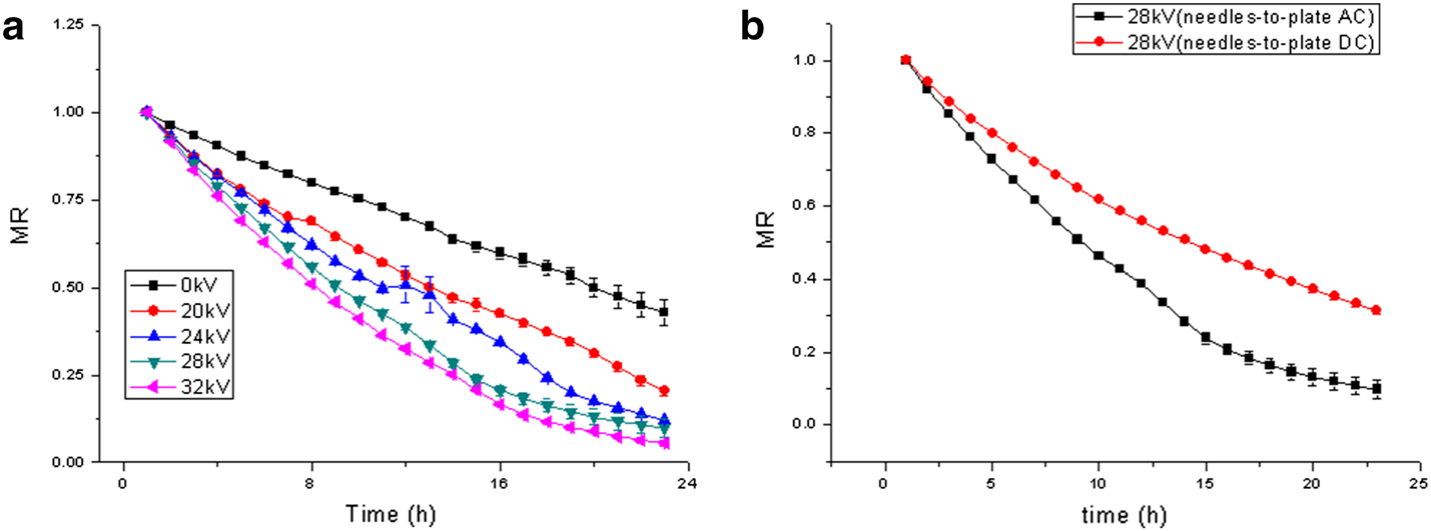
Variation of moisture ratio of Chinese wolfberry fruits. **a** Different voltages, **b** different electrodes

Figure 4 shows the moisture ratio curve of Chinese wolfberry fruit samples treated with different voltage values. It is evident that the moisture ratio of Chinese wolfberry fruit samples was significantly reduced under EHD compared with that of control. The more voltage values are higher, the more the moisture ratio reduced faster.

By ANOVA, the results showed that the moisture ratio of Chinese wolfberry treated at 20, 24 and 28 kV with multiple needles-to-plate electrode under AC electric field have statistically significant difference (*p* < 0.05) and very statistically significant difference at 32 kV (*p* < 0.01) compared to control.

Figure 4b shows that the comparison of moisture ratio of Chinese wolfberry treated by EHD at 28 kV with multiple needles-to-plate electrode under AC electric field and multiple needles-to-plate electrode under DC electric field. By ANOVA, the results showed the moisture ratio values have statistically significant difference (*p* < 0.05) for each drying conditions.

### Rehydration ratio

**Fig. 5:**
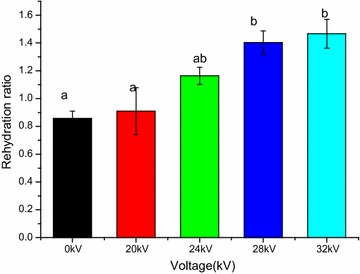
Effect of different voltages on rehydration ratio of dried Chinese wolfberry fruits. For each response, means with *different lower case letters* are significantly different (*p* < 0.05)

Figure 5 shows that the effect of different voltage on rehydration ratio of dried Chinese wolfberry fruit samples. ANOVA results showed that Chinese wolfberry fruits exposed to EHD process had a significant effect on rehydration ratio compared to control (*p* < 0.05), this result is in consistent with the consequence discovered by Esehaghbeygi et al. (2014), they reported that the treatment used had a significant effect on the rehydration ability of the materials at 6 and 8 kV cm^−1^, but no significant effect on the final shrinkage at 6 and 8 kV cm^−1^. As seen from Fig. 5, the rehydration ratio of Chinese wolfberry fruits dried by EHD was higher than that of control and the rehydration ratio increased with the increase of the voltage. Some authors reported that the EHD treated materials exhibited higher rehydration ratio (Dinani, and Havet 2015), this result is in agreement with the present study. But bai et al. (2013) reported the rehydration ratio by the EHD-dried sample was 46.77/100 g, which is 9.52/100 g more than the rehydration ratio by oven dried materials, on a dry weight basis, in contrast the final shrinkage values for EHD was significantly lower than that for oven drying. In addition Dinani and Havet (2015) explained that faster drying, along with higher voltage, caused the sample to develop a porous structure, increasing its ability to absorb a higher amount of water during rehydration. Therefore the relationship between rehydration capacity and shrinkage is very complex. The reasons of the higher rehydration capacity of the dried Chinese wolfberry fruit samples dried at higher voltages are that more water is absorbed by the highly porous structure and they remains lower moisture content (Bai et al. 2013).

### Shrinkage

**Fig. 6:**
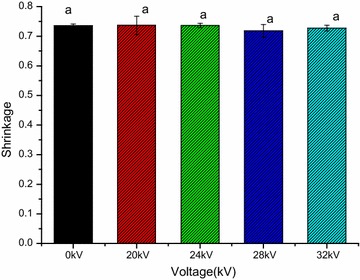
Effect of different voltage on shrinkage of dried Chinese wolfberry fruits. For each response, means with *different lower case letters* are significantly different (*p* < 0.05)

From Fig. 6, it is clear showed that all the treatments had no significant difference on shrinkage of dried Chinese wolfberry fruits (*p* > 0.05). The shrinkage percentage of dried at 0, 20, 24, 28 and 32 kV was 73.581, 73.665, 73.624, 71.834 and 72.76 %, respectively. In other word, no significant different for shrinkage was observed in treated samples. This result is in consistent with the consequence discovered by Bai et al. (2012), they reported that the final shrinkage values for EHD and ambient air drying were 63.70 and 65.81 %, respectively. The reason they explained is that the less shrinkage might be associated with the organizational structure of material. In addition, similar results had been obtained for tomato slices (Esehaghbeygi and Basiry 2011) and Esehaghbeygi et al. (2014) dried banana slices by electrohydrodynamic and microwave and the conclusion is consistent with our result. They illustrated that no significant differences were observed in the shrinkage of EHD at 6, 8 and 10 kV cm^−1^. In addition, Singh et al. (2013) discovered that there is no shrinkage in potato slices pre-treated with a high electric field. They explained that this characteristic can be attributed to the effect of the high electric field, under the influence of the electric field, the cell membranes may have permeabilized and water was evenly distributed throughout the sample and all of these changes lead to a uniform drying and less shrinkage. Shrinkage seems to play an important role in drying Chinese wolfberry fruits. Shrinkage may be related to structural properties of materials and different drying treatments. The Chinese wolfberry fruits have a special wax coat structure, both this structure and the influence of multiple needles-to-plate electrode under AC electric field on the Chinese wolfberry fruits may lead to no significant different for shrinkage.

### Vitamin C contents

Table 3 shows that Vitamin C contents of the dried Chinese wolfberry fruits and drying time which for the Chinese wolfberry fruits were dehydrated until they reached the final moisture content (17 ± 1)/100 g. Vitamin C contents can indicate physical and chemical changes in the samples during drying because it is easy to degenerate. As seen from Table 3, Vitamin C contents of the dried Chinese wolfberry fruits exposed to EHD are higher than that of control, together with the Vitamin C contents of the dried Chinese wolfberry fruits increased with the reduction in drying time and the high voltage electric field did not destroy Vitamin C. By ANOVA, the results showed that the Vitamin C contents of Chinese wolfberry treated at 20 and 24 kV with multiple needles-to-plate electrode under AC electric field have statistically significant difference (*p* < 0.05) and very statistically significant difference at 28 and 32 kV (*p* < 0.01) compared to control.

**Table 3:** Vitamin C contents of dried Chinese wolfberry fruits

Test sample	0 kV	20 kV	24 kV	28 kV	32 kV
Drying time (h)	49	31	28	25	22
Vitamin C contents (mg/100 g)	29.9 ± 3.4^a^	35.3 ± 2.0^b^	35.2 ± 0.4^b^	37.3 ± 2.6^c^	36.9 ± 1.4^c^
Final moisture content	(17 ± 1)/100 g				

Data are shown as the mean ± standard deviation (SD). For each response, means with different lower case letters are significantly different (*p* < 0.05)

### Specific energy consumption of EHD drying

**Fig. 7:**
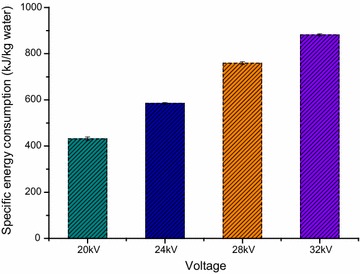
The specific energy consumption of EHD drying (kJ·kg^−1^ water) versus different alternating voltage

From Fig. 7 one could see that the specific energy consumption of EHD drying (kJ·kg^−1^ water) were significantly influenced by the alternating voltage (*p* < 0.05) and the energy required to evaporate 1 km of water increased with an increase in alternating voltage. This relationship is in consistent with the consequence summarized by Singh et al. (2012).

The total specific energy consumption (TSEC_EHD_) of the electric power of all used equipment which contains AC/DC high voltage power source and voltage regulator at 32 kV multiple needles-to-plate electrode under AC electric field treatment, the specific energy consumption of EHD drying (SEC_EHD_) for 32 kV multiple needles-to-plate electrode under AC electric field treatment are showed in Table 4. The energy efficiency was calculated by values of the specific energy consumption of EHD drying (SEC_EHD_) and the total specific energy consumption (TSEC_EHD_). It is easy calculated that the energy efficiency is 22.49 %, the result is higher than that of Martynenko and Zheng (2016). The reason may they applied more complex equipment.

**Table 4:** Specific energy consumption at different drying regimes

Calculated type	SEC_EHD_	TSEC_EHD_
Value (kJ·kg^−1^)	882	3920

EHD drying consumed the total energy of 3920 kJ to evaporate one kilogram of water, whereas hot air drying required 22,902 kJ, the value came from database of hot air drying Chinese wolfberry fruits in the local drying industry. EHD drying expanded only 17.12 % of the energy need for hot air drying, in other words the energy hot air drying consumed is 5.8 times as much as that EHD drying required. This is in agreement with previous analysis of energy used up in EHD drying and oven drying (Bai et al. 2012).

### Selection of the best mathematical model

**Fig. 8:**
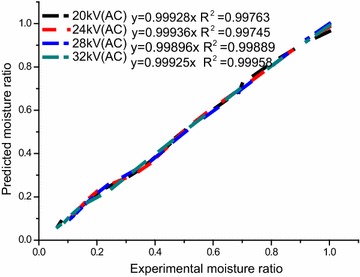
Comparison of experimental values with predicted values of moisture ratio at different voltages using the Parabolic model

To obtain the superior mathematical models of Chinese wolfberry fruits, non-linear regression analysis was carried on to estimate the constants and parameters of the ten drying mathematical models given in Table 1. The coefficients of drying models and the comparison criteria that contains the root mean square error (E_RMS_), reduced mean square of the deviation (*χ*^2^) and the coefficient of correlation (*R*^2^) from mathematical models is given in Table 5. Those parameters are used to evaluate goodness of fit for different drying curves. The best model describing the drying characteristics of Chinese wolfberry fruits was chosen as the one with the highest *R*^2^ values and the lowest *χ*^2^ and E_RMS_ values (Doymaz 2012). Table 5 distinctly shows that the values of *R*^2^, *χ*^2^ and, E_RMS_ of Lewis (Newton), Henderson and Pabis, Logarithmic, Parabolic, Page, Dinani et al., Wang and Singh, Modified Page, Midilli et al., and Weibull. In addition, the average values of *R*^2^, *χ*^2^ and E_RMS_ for different models (Table 6) were from 0.959753 to 0.998255, 0.00014 to 0.00309 and 0.010643 to 0.05315, respectively. It is clear that due to *R*^2^ of all ten mathematical models are above 0.95, accounting for the ten models could satisfactorily describe drying curves of Chinese wolfberry fruits treated by EHD. Of all the models fitted, the Parabolic model give the highest values of *R*^2^ and the lowest values of *χ*^2^ and E_RMS_. Accordingly, the Parabolic model were selected as the best models to represent the drying behavior of Chinese wolfberry fruits by EHD drying technology. In addition, validation of the selected models was determined by comparing the predicted values with the experimental values of moisture ratios at different levels of voltages (Dinani et al. 2014; Tahmasebi et al. 2014). The comparison of experimental values with predicted values of moisture ratio at different voltages using the Parabolic model can be seen in Fig. 8. It is clear that the data are mainly scattered adjacent to the 45°-straight line, which indicated that the Parabolic model could reflect the drying kinetics and characteristic of Chinese wolfberry fruits perfectly. Therefore, the Parabolic model was determined to be the best for describing the drying characteristics of Chinese wolfberry fruits.

**Table 5:** Results of statistical analyses on the modeling of moisture ratio and drying time

Model	Voltage (kV)	*k*	*n*	*a*	*b*	*c*	*R*_*MES*_	*R* ^2^	*χ* ^2^
Lewis	20	0.0560					0.040122	0.95891	0.001677
	24	0.0698					0.056947	0.95697	0.003378
	28	0.0838					0.055390	0.96203	0.003208
	32	0.0949					0.060140	0.96110	0.003774
Henderson and Pabis	20	0.0663		1.1042			0.041772	0.97396	0.001869
	24	0.0807		1.1134			0.038037	0.97997	0.001573
	28	0.0981		1.1546			0.028874	0.98892	0.000913
	32	0.1118		1.1721			0.029503	0.98892	0.000953
Logarithmic	20	0.0280		1.6729	−0.6566		0.013737	0.99766	0.000202
	24	0.0398		1.5519	−0.5009		0.013858	0.99722	0.000218
	28	0.0680		1.2979	−0.2025		0.014653	0.99700	0.000247
	32	0.0789		1.2828	−0.1768		0.010262	0.99862	0.000121
Parabolic	20			1.0078	−0.0440	0.00042	0.013389	0.99746	0.000198
	24			1.0409	−0.0572	0.00075	0.013861	0.99722	0.000218
	28			1.0750	−0.0766	0.00146	0.009369	0.99879	0.000101
	32			1.0729	−0.0835	0.00170	0.005954	0.99955	0.000041
Page	20	0.0229	1.3407				0.027380	0.98925	0.000801
	24	0.0261	1.3768				0.018660	0.99364	0.000378
	28	0.0334	1.3713				0.012251	0.99801	0.000164
	32	0.0376	1.3837				0.015743	0.99812	0.000271
Dinani et al.	20			1.1815	−11.0882	26.0414	0.021580	0.99340	0.000515
	24			1.3111	−11.4165	22.8014	0.017829	0.99540	0.000361
	28			1.6849	−14.8427	21.7420	0.010167	0.99856	0.000119
	32			1.7242	−13.6181	19.6121	0.007640	0.99917	0.000076
Wang and Singh	20			−0.0430	0.000394		0.013610	0.99747	0.000198
	24			−0.0508	0.000539		0.018709	0.99516	0.000380
	28			−0.0638	0.001010		0.024673	0.99192	0.000667
	32			−0.0711	0.001260		0.022994	0.99343	0.000579
Modified Page	20	0.0598	1.3407				0.028029	0.98925	0.000839
	24	0.0708	1.3768				0.021434	0.99364	0.000499
	28	0.0839	1.3713				0.012248	0.99801	0.000164
	32	0.0934	1.3837				0.012287	0.99812	0.000165
Midilli et al.	20	0.0758	0.7024	1.0859	−0.01392		0.011391	0.99809	0.000149
	24	0.0550	0.9703	1.0605	−0.00934		0.013909	0.99707	0.000230
	28	0.0416	1.2852	1.0267	−0.00068		0.011442	0.99808	0.000158
	32	0.0589	1.1862	1.0541	−0.00226		0.008520	0.99900	0.000088
Weibull	20			1.3407	16.72450		0.028029	0.98925	0.000840
	24			1.3768	14.13300		0.021433	0.99364	0.000499
	28			1.3713	11.91583		0.012248	0.99801	0.000164
	32			1.3837	10.70975		0.012287	0.99812	0.000165

**Table 6:** Average values of *χ* ^2^, E_RMS_ and *R* ^2^ for used models of needle-plate electrodes alternating voltage

Parameters	Lewis	Henderson	Logarithmic	Parabolic	Page	New model	Wang	Midilli	Modifie	Weiull
*χ* ^2^	0.00301	0.00133	0.00020	0.00014	0.00040	0.00268	0.00046	0.00016	0.000412	0.00041
E_RMS_	0.05315	0.03455	0.01313	0.01064	0.01851	0.01430	0.01999	0.01132	0.0185	0.01850
R^2^	0.95975	0.98294	0.99763	0.99826	0.99476	0.99663	0.99450	0.99806	0.99476	0.99476

The generalization of different selected models against defined variables was used by some researchers (Dinani et al. 2014; Tahmasebi et al. 2014). In the present study, the multiple combinations of different parameters that gave the highest *R*^2^ were included in the generalized Parabolic model. In order to explain the influence of the drying variables on the Parabolic model, the values of *a*, *b* and *c* parameters were regressed against the voltage (V) in kV using multiple regression analysis. The constants and coefficients of the generalized model were as follows:

16 $${\text{MR}} = a + bt + ct^{2}$$

17 $$a = 1.58448 - 0.00756{\text{V}} - 8.54463{\text{V}}^{ - 1} \quad R^{2} = 0.966157$$

18 $$b = - 0.08713 - 0.00128{\text{V}} + 1.39066{\text{V}}^{ - 1} \quad R^{2} = 0.979457$$

19 $$c = - 0.00091 + 0.00009{\text{V}} - 0.01194{\text{V}}^{ - 1} \quad R^{2} = 0.964552$$

**Fig. 9:**
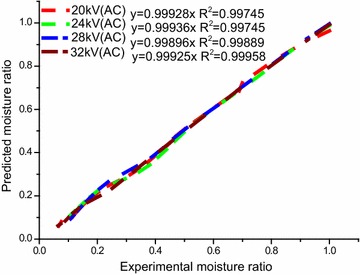
Comparison of experimental moisture ratio with predicted moisture ratio of Chinese wolfberry fruits at different EHD drying treatments from the generalized Parabolic model

The generalized Parabolic model indicates consummate accuracy for the estimation of the moisture ratio of Chinese wolfberry fruits at any time during the drying process. Figure 9 shows the comparison of the experimental and predicted moisture ratio values by the generalized Parabolic model at different voltages. The results of statistical analyses on the generalized Parabolic modeling of moisture ratio and drying time of multiple needles-to-plate electrode under AC voltages are showed in Table 7. The results show that the values of *R*^2^ of the generalized Parabolic drying models were ranged from 0.994331 to 0.999233, *χ*^2^ and E_RMS_ values were ranged from 0.00015958 to 0.00027234 and 0.01177991 to 0.01548101, respectively, indicating the evident consistency of the model and relationship between the coefficients and drying variables. The most important advantage of using this generalized equation is being independent from different parameters of *a*, *b*, *c* coefficients for different drying treatments (Dinani et al. 2014). Therefore, it can be stated that the generalized Parabolic model gives an adequate description of the EHD drying of Chinese wolfberry fruits at different voltages.

**Table 7:** Results of statistical analyses on the generalized Parabolic modeling of moisture ratio and drying time of multiple needles-to-plate electrode under AC voltages

Voltage (kV)	*R* ^2^	E_RMS_	*χ* ^2^
20	0.994331	0.01374245	0.00020909
24	0.998492	0.01548101	0.00027234
28	0.999233	0.01177991	0.00015958
32	0.997553	0.01273842	0.00018545

## Conclusion

1. The electrohydrodynamic (EHD) method can obviously strengthened the drying rate of Chinese wolfberry fruits. The drying rate Chinese wolfberry fruits exposed to EHD process increased with strengthen of applied voltage and also the mass transfer enhancement factor heightened with the increase of voltage. By the comparison of current and the mass transfer enhancement factor of the drying process treated by EHD at 28 kV with multiple needles-to-plate electrode under AC electric field and multiple needles-to-plate electrode under DC electric field, the type of electric field had a most significant effect on enhancing the drying rate.
2. Higher electric field led into a higher level of moisture removal, rehydration ratio and content of Vitamin C of Chinese wolfberry fruits, compared to control. Also, the remaining moisture content, rehydration ratio and content of Vitamin C increased with an increase of applied voltage. But, no significant difference on shrinkage of dried Chinese wolfberry fruits was found compared to control.
3. The increase of voltage increased the specific energy consumption (SEC_EDH_) and the energy efficiency is 22.49 %, so energy consumption at EHD drying was mostly determined by the efficiency of AC/DC high voltage converter and voltage regulator. Compared to hot air drying, EHD drying expanded only 17.12 % of the energy need for hot air drying. Therefore, EHD drying for Chinese wolfberry fruits is more efficient in terms of energy saving.
4. By the root mean square error (E_RMS_), reduced mean square of the deviation (*χ*^2^) and the coefficient of correlation (*R*^2^), the Parabolic model was found to be suitable for describing the drying characteristics of Chinese wolfberry fruits under different voltages. So, due to the advantages of EHD drying method of Chinese wolfberry fruits, this method can be offered as an improved method for drying Chinese wolfberry fruits.

## Abbreviations

EHD: electrohydrodynamic
AC: alternating current
DC: direct current
MR: moisture ratio

### List of symbols

*c*: the water vapor concentration
*c*_0_: water vapor concentration at the sample surface (kg m^−3^)
*c*_*∞*_: water vapor concentration in air (kg m^−3^)
D: mass diffusivity (m^2^ s^−2^)
d: diameter of the wire electrode (m)
Δ*c*: the difference in water vapor concentration between the sample surface and ambient
h_m_: mass transfer coefficient (m s^−1^)
I: corona current (μA)
$$\dot{m}$$
mass transfer rate (g h^−1^)
*ρ*: density of air (kg m^−3^)
*φ*: relative humidity
*P*: atmospheric pressure (kPa)
*P*_*g*_: saturated pressure of water vapor (kPa)
Sh: sherwood number
*χ*^2^: reduced mean square of the deviation
E_RMS_: root mean square error
*R*^2^: coefficient of correlation
